# Affective priming through Indian ragas: influence on perception of ambiguous visual stimuli and creativity

**DOI:** 10.3389/fpsyg.2026.1723673

**Published:** 2026-01-29

**Authors:** Alisha Deen

**Affiliations:** Chitkara School of Psychology and Counselling, Chitkara University, Chandigarh, Punjab, India

**Keywords:** affective priming, alternative uses task, Charukeshi, creativity, Indian ragas, Kedar, perception, Torrance Tests of Creative Thinking – Figural

## Abstract

Indian ragas have captivated a sustained scholarly interest over the past few years owing to their capacity to evoke emotional, therapeutic and physiological benefits. A conspicuous research gap persists: ragas have seldom been examined as an affective prime; their unconscious influence on subsequent perception, behavior, and creativity remains unexplored and antecedent research into music and creativity has predominantly been confined to background music only. This research endeavored to ascertain whether ragas of two contrasting emotional valences of happiness and sadness unconsciously influence the emotional expression and perception congruent with the primed affective tone, while also bolstering divergent thinking. Participants (*n* = 90) were randomly assigned to an experimental (*n* = 45) or a control cohort (*n* = 45). The experimental cohort underwent a counterbalanced, within-subjects exposure to Raga Kedar (positively valenced) and Raga Charukeshi (negatively valenced), whereas the control group enabled a between-subjects contrast. As measured by the Emotional Word Selection Task to describe the ambiguous images, the findings elucidated a striking congruence between the lexical choice and the affective tone, where Raga Kedar primed more positive word choices and Raga Charukeshi primed more negative word choices, in contrast to the control cohort, which evinced an affective neutrality. This provides stark evidence for top-down processing of perception. The creativity indexed by performance on the Alternative Uses Task and the Torrance Tests of Creative Thinking – Figural demonstrated an augmentation of creative output not only from the priming effect of positive music, though exerting a more pronounced effect, but also from negative music, and the control cohort showcased lower creativity scores compared to the experimental conditions, which highlights the plausibility of a dual-pathway model of creativity due to the priming effect of both positive and negative music in facilitating creativity.

## 1 Introduction

Music is an integral part of human existence. As one of the first stimuli we register upon waking up, it permeates even through sleep inertia (McFarlane et al., 2020), whether through the chirping of birds or the music of the alarm. It influences not just our conscious perception but also unconscious emotional processing (Kim, 2023). Since time immemorial, music has been our constant companion, consciously used by people to relax, self-regulate their mood and emotions, change emotions, express emotions, or to match their current emotions (Juslin, 2013; Stewart et al., 2019). Hence, the positive and negative valence of music has been used ever since to give meaning to our lives. Extant literature demonstrates that music increases positive affect and reduces negative affect, influences health and well-being, alleviates anxiety and depression in both depressed and non-depressed individuals, making it an indispensable part of human existence (Sakka and Juslin, 2017; Dingle et al., 2021; Blasco-Magraner et al., 2023).

The concept of emotional contagion was pioneered by Hatfield et al. (1994) in social psychology, which was later extended to music by Juslin and Västfjäll (2008), accentuating the underlying mechanisms that make music contagious. Music functions as a powerful conduit of emotions, deriving its power from the perception of people, how they perceive music as expressing some emotions, and it induces the same emotions in them (Lundqvist et al., 2009; Lauria, 2023). Perceiving upbeat rhythms of drums at a wedding as happy may make you feel happy, or perceiving a funeral song as sad may make you feel sad or perceiving the hymns sung at a temple as peaceful may make you feel calm and so on. This phenomenon occurs due to the mirror neurons in the premotor cortex and the right inferior frontal gyrus, which enable internal simulation of perceived emotions and emotions felt, mirroring the emotions perceived, hence the listeners feel the same emotion as they perceive from the music (Molnar-Szakacs and Overy, 2006; Proverbio and Zani, 2022).

The most cited reasons for why people listen to music have been credited to its ability to induce strong emotions in them (Juslin and Laukka, 2004). Researchers have long been perplexed by this paradox: despite being unlike other stimuli like threats of dangerous animals, which have been carried on with the human race through evolution, accounting for their intrinsic survival value, music does not have any intrinsic survival value of its own – yet it induces strong emotions in humans (Juslin and Västfjäll, 2008; Honing et al., 2015; Trainor, 2015). There has not been a consensus among scholars on the definition of emotions, but a common ground exists regarding the experience and expression of emotions. Hence, this study employed the ‘circumplex model of affect’, analyzing only the valence aspect (Russell, 1980). Its quantification and measurement make it a favorable choice for testing affective states (Remington et al., 2000). The research at hand drew 28 affective words from the ‘circumplex model of affect’ for the Emotional Word Selection Task.

Now consider this scenario: an individual is driving their car, and upon switching on the stereo, a happy song starts playing. Earlier, they could not wait for the journey to end, but now with the music, they start enjoying the ride. Their body starts grooving to the beats of the music without conscious effort, their thoughts naturally align with the emotional valence of the music, and that road rage they often felt disappears. A single stimulus unconsciously influenced not just their emotions but also their subsequent behavior. This phenomenon is called affective priming, illustrating how an emotionally charged stimulus, such as music, influences our behavior toward the subsequent stimulus without the individual being consciously aware of it.

The genesis of the concept of semantic priming was laid out in a seminal paper by Meyer and Schvaneveldt (1971), which elucidated that exposure to a prime accelerated faster and more accurate responses to the target stimulus. This concept was further extended to music being an affective prime by Steinbeis and Koelsch (2011), whose findings harbored significant implications that participants responded more quickly when the affect of musical primes was congruent with the subsequent target word compared to incongruent pairings. Within the realm of music, another language-based study was done by Tay and Ng (2019), who employed lexical choice to demonstrate that affective priming through music dictated the participants’ linguistic choices, where the emotional words selected were congruent with the valence of the music.

Instead of being limited to the use of language, the impact of affective priming also extends to the perception of visual stimuli like facial expressions, whether neutral or ambiguous. Prior research by Logeswaran and Bhattacharya (2009) gave conclusive evidence using ERP machines that neutral faces were rated as happier when preceded by happy music, which acted as an affective prime. Adding to this corpus, Huziwara et al. (2022) also yielded discernible evidence in favor that consonant (happy) and dissonant (sad) musical chords expedited facial expression recognition for congruent facial emotions. Encapsulating these findings, it is posited that music acts as a powerful affective prime that alters not just the perception of neutral or ambiguous visual stimuli, but also the lexical choices of the participants to describe that visual stimulus – a relationship investigated in the present study.

Priming is categorized based on conscious awareness into subliminal and supraliminal priming. Subliminal priming means below the consciousness threshold, implying that the stimulus is processed unconsciously without awareness, while supraliminal priming means above the consciousness threshold, connoting that the stimulus is perceived consciously with awareness and yet exerts an unconscious influence on subsequent behavior. Most of the investigations on priming since long back embedded verbal messages subliminally under music rather than subliminal music below the conscious level (Kaser, 1986; Egermann et al., 2006). Recent research, which aimed to investigate the priming effects via music, exclusively incorporated supraliminal priming through music, showcasing a significant effect of music on subsequent mood and perception; however, only when music was heard consciously, even if passively or incidentally (Lundqvist et al., 2009; Steinbeis and Koelsch, 2011; Tay and Ng, 2019).

To date, there exists no concrete empirical evidence in favor of music functioning as a true subliminal prime; rather, music needs to be consciously heard for incurring a reliable priming effect. Increased awareness regarding the respective prime maximizes the priming effect, which pinpoints that the degree of perceptual awareness plays a pivotal role in accentuating the strength and reliability of priming effects (Lohse and Overgaard, 2019). In day-to-day life, music is hardly, if ever, heard subliminally. Audible music is often heard consciously by almost everyone, be it while driving, watching television or exercising. Hence, Indian ragas as supraliminal primes reflect everyday experiences with music, increasing the ecological validity along with perceptual clarity of the research.

A broader and more integrated activation of the cortical network is observed in supraliminal priming, as compared to subliminal priming. Previous research expounds a widespread engagement of parietal regions responsible for attention and posterior visual regions, inferior frontal areas involved in language and semantic integration when the primes were consciously perceived (Kouider et al., 2006; Heinzel et al., 2008). Through fMRI findings, Meneguzzo et al. (2014) discovered that supraliminal priming increases the activation of left rostral anterior cingulate, a region attributed to higher-order functions like emotional regulation, conscious cognitive evaluation and error detection of the conscious stimuli, which crosses the awareness threshold, thereby engaging top-down perception. The converging evidence from these findings underscores that supraliminal priming stimulates and engages the distributed neural network responsible for conscious, emotionally grounded and flexible cognitive processing.

Affective priming proffers a plausible evidence for top-down processing as expounded by Jolij and Meurs (2011), that mood induced via music biased visual perception, wherein the participants perceived emotional faces even in noisy images which were congruent with the mood induced by the music (happy or sad) and even reported illusory faces in the absence of any face. In harmony with this, Zhang and Asano (2024) also postulated a congruence between music and visual stimuli, which contributes toward the perceptual immersion. Bringing to the fore, these findings articulate perception as a process of active construction shaped by conscious and unconscious processes and the emotional context. Rebutting the bottom-up view, which propounds perception as only driven by the external stimuli, these findings elucidate robust evidence for the top-down model of perceptual processing, stating that our sensory experiences are an amalgamation of external stimuli, prior experiences, knowledge, expectations, goals and emotional states. In a similar vein, this study is grounded in the premise that music as an affective prime can bias the perceptual processing, hence presenting a strong case in favor of top-down processing.

Cohesively, these studies confer credence for music being an affective prime that modulates the lexical choices and perception of ambiguous visual stimuli, which is in line with what this study aims to evaluate, but through a different genre of music, which has not yet been tested as an affective prime, i.e., Indian ragas. Indian classical music comprises two major traditions: Hindustani classical music (HCM) of Northern India, and Carnatic classical music of South India. The present study focuses on the ragas within HCM, where ‘ragas’ were mentioned in ancient Vedic texts of *Natya Shastra* (c. 2nd century BCE). Ragas, being the heart and soul of the Indian culture and music, derive their origin from a Sanskrit term “ranj” which means “colour” or “mood” (Ranade, 2006). As defined by Pudaruth (2016), “a raga is a specific melodic configuration produced out of a group of notes rendered systematically and beautifully.” Unlike many other genres of music, HCM is characterized by complex structures and a long historical tradition, and it will help shed light on the phenomena of priming through music uniquely. The masterpieces of HCM consist of tune patterns known as ragas, which are famous for their ability to evoke and communicate specific and diverse moods, such as joyful, serene, or uplifting, as well as melancholic, introspective, or tense qualities (Valla et al., 2017).

Beyond tradition, recent empirical studies validate that tonal features, such as major versus minor notes, which correspond to shuddh versus komal notes in HCM, reliably evoke positive and negative affect in the ragas (Mathur et al., 2015). In therapeutic contexts, exposure to ragas or raga therapy has been linked to alleviating stress, depression and anxiety. A pilot study by Tripathi et al. (2022) postulated that listening to raga reduced stress and significantly helped in stress management. Recent empirical work by Chand et al. (2024) reported a reduction in depression and anxiety after exposure to Raag Bhairavi. Another recent research by Chaki et al. (2024) found that happy and sad HCM as an immediate intrinsic musical context influences the perception of emotions evoked by music.

A growing body of research elucidates that ragas modulate not just mood and stress, but also the neural activity that aligns with their affective associations. Tangible evidence provided by Babel et al. (2023) via EEG findings suggests that listening to ragas causes changes in arousal, attention, and emotional regulation. A recent EEG study by Sahoo et al. (2025) discovered that ragas with major or shuddh notes elicit positive emotion states like joy or calmness and displayed greater cortical engagement, whereas ragas with minor or komal notes elicited negative emotional states like sadness or tension and lower arousal. In a similar vein, Raga Kedar has all major or shuddh notes except the note of ‘ma’ as it’s both shuddh (major note) and tivra (sharp note), hence it is associated with positive emotions. Raga Charukeshi uses five major or shuddh notes and two minor or komal notes, which are dha and ni, associated with negative emotions. As a departure from the previous studies, this research uses ragas as an affective prime, as they have been proven in existing research to be a powerful conduit for emotions. This study seeks to bridge the cultural-musical insights with psychological research on priming, thereby exploring the potential of these culturally rich and affective music genres to test whether or not they unconsciously shape perception and catalyze creativity.

Earlier empirical work credits music for not just being potent in orchestrating an impact on our emotional states but also on the subsequent cognitive processes, including creativity. Creativity is defined as “the genesis of original and novel socially applicable ideas, insights, or solutions to problems” (Sternberg and Lubart, 1996; Fink et al., 2010). It was unveiled by Ritter and Ferguson (2017) that listening to happy, arousing music stimulates higher divergent thinking in comparison to the absence of music. Research by Hosseini et al. (2019) also found an enhanced creativity performance on the alternative uses creativity task after exposure to positive background music, thereby validating music as an influential environmental factor and a prominent emotional catalyst for cognitive processing, with consequent ramifications for creativity.

Earlier research on creativity predominantly postulated that only positive music increases creativity, but diverging from this, recent studies endorse that both positive and negative music bolster creativity and suggest a “dual-pathway model” of creativity (Xiao et al., 2023). Creativity is enhanced via positive and negative affective music, proposing a “dual-pathway model,” which outlines that both positive and negative emotions take distinct pathways for increasing creativity. According to this dual-pathway model, positive music broadens the attentional scope, leading to enhanced cognitive flexibility and accelerated information processing, which in turn increases fluency and originality. On the other hand, negative music fosters creativity by narrowing the attentional focus, which in turn preserves the cognitive resources and maintains moderate arousal. The hypothesis that positive music broadens the attentional scope is explicated by another phenomenal research by Fredrickson (2001), who unearthed the concept of “Broaden-and-Build theory,” which maps out that positive emotions broaden the cognitive repertoires of the individual, which helps them in generating original and innovative ideas, thereby fostering higher creative output.

These accumulated studies employed background music for bolstering creativity, whereas the current study operationalized priming through music that too, via Indian ragas for augmenting creativity, which adds to the repertoire of research that priming through music enhances divergent thinking and figural creativity. As a departure from past research, which largely focused on positive music, the present study examines whether both positive and negative music in the genre of ragas can serve as affective primes that bolster creativity, filling a key gap in the literature and thereby extending the dual-pathway perspective into the domain of Indian classical music.

## 2 Materials and methods

### 2.1 Participants

A total of 100 participants were initially recruited for the study. The study incorporated convenience sampling for data collection, wherein participants were selected based on their availability and willingness to participate. Participants recruited in the study were school teachers, healthcare workers, and individuals working in government and private institutions. The objective was to understand the influence of music on the general adult population with no musical experience. All participants were asked to fill in the consent form before participating in the research. Inclusion criteria were adults ranging from the age group of 25–55 years without any hearing loss or impairment, and without a formal musical training or background, to avoid bias from prior musical expertise. Exclusion criteria included a history of neurological or psychiatric conditions, e.g., major depression, schizophrenia, PTSD, current use of psychoactive medications or diagnosed with any hearing impairments or having a formal musical training or background. The participants filled in a pre-experimental online questionnaire to screen them based on the inclusion and exclusion criteria and familiarity with Indian classical music and the specific ragas used in the study, as prior familiarity with the ragas could introduce a potential bias. Before the main experiment, all participants completed an online Positive Affect and Negative Affect Schedule (PANAS) to assess mood and emotional valence. Based on PANAS results, six participants were excluded due to abnormal mood valence or elevated levels of sadness, which could have influenced the study’s outcomes. Out of the remaining 94 participants, three were excluded due to not comprehending the Alternative Uses Task (AUT)—one gave only the basic function of an umbrella, and two provided literary compositions—and one participant did not complete the Torrance Tests of Creative Thinking - Figural. Following these exclusions, the final sample used for analysis consisted of 90 participants.

The 90 participants were equally divided based on gender (45 males and 45 females; *M* = 1.5, SD = 0.50), which were further categorized into three age groups: 25–35 years (*n* = 30), 36–45 (*n* = 30), and 46–55 (*n* = 30). Participants within each age group were randomly and equally assigned to either the experimental group (*n* = 15) or the control group (*n* = 15), resulting in a total of 45 participants in both the experimental and control groups.

#### 2.1.1 Ethics approval

The study involving human participants was reviewed and approved by the Institutional Human Ethical Committee (IHEC), Chitkara University, Punjab (Ethics Committee Registration No. EC/NEW/INST/2022/PB/0081). The study was formally approved under the Approval no. EC/NEW/INST/2025/531/409. The participants provided their written informed consent to participate in this study.

### 2.2 Music stimuli

Supraliminal affective priming was undertaken via two ragas, i.e., Raga Kedar and Raga Charukeshi. A 12-min music excerpt of an instrumental piece without lyrics of Raga Kedar was extracted from the music piece performed by Purbayan Chatterjee (sitar) and Shubh Maharaj (tabla), performance available on YouTube.^1^ Similarly, a 12-min music excerpt of an instrumental piece without lyrics of Raga Charukeshi was extracted from the music piece performed by Ustad Shahid Parvez Khan (sitar) and Akram Khan (tabla), available on YouTube.^2^ Due to copyright restrictions, this audio file cannot be provided with the article or Supplementary material. These ragas were specifically chosen as they efficiently represent two distinct valences, where Raga Kedar had a positive affect, and Raga Charukeshi had a negative affect. Ragas inherently involve tempo variation as part of their expressive structure; therefore, the 12-min musical excerpts began with a slow, non-metric introduction and gradually developed, reaching approximately 120 bpm for the positive raga and 60 bpm for the negative raga. In order to ascertain the reliability and validity of the respective valence associated with the ragas, 12 school teachers were asked to rate the emotional valence of the ragas. Consequently, these ragas representing positive and negative valences were validated through these relevant ratings.

### 2.3 Measures

#### 2.3.1 PANAS scale

In the preliminary stages preceding the formal experiment, all participants completed an online Positive Affect and Negative Affect Schedule (PANAS) to assess their mood and emotional valence. In the present study, the scale was used to measure how the participant had been feeling over the week. The PANAS was administered to exclude individuals with abnormal mood valence or elevated levels of sadness, which could have influenced the study’s outcomes. After completing PANAS, participants filled in a questionnaire, where they filled in their demographic details relevant to the inclusion and exclusion criteria of the study.

PANAS is an extensively employed 20-item self-report measure intended to assess the two prominent orthogonal dimensions of mood: positive affect (PA) and negative affect (NA)—as originally proposed by Watson et al. (1988). Accordingly, it was used in the study to quantify the prior mood of the participants before the experiment. In PANAS, the participants are presented with 20 words describing different feelings and emotions, out of which 10 words represent the positive affect (e.g., interested, excited, strong, enthusiastic, proud) and 10 words represent the negative affect (e.g., distressed, upset, guilty, nervous, afraid). For each word, participants indicate to what extent they feel that emotion using a 5-point Likert scale, typically ranging from 1 (very slightly or not at all) to 5 (extremely). The scale can be framed to measure affect “right now” (state affect), “today,” “past week,” “past few weeks,” or “in general” (trait affect), depending on the research goal. Various studies expound the robustness and high internal consistency reliability of the measure, with Cronbach’s alpha (*α*) values as α = 0.85 for PA and α = 0.83 for NA (Estévez-López et al., 2016; Kumar et al., 2025). Owing to the construct validity of the two-factor model of the scale, which is substantiated by a wealth of empirical evidence, propagating its robustness across various samples, makes it a reliable tool to be employed in the study for mood metrics (Wedderhoff et al., 2021).

#### 2.3.2 Emotional Word Selection Task (EWS)

In the Emotional Word Selection Task, participants are instructed to choose three words out of a pool of words to describe the emotional valence of each ambiguous picture. Initially, a pool of 28 words was drawn from the circumplex model of affect given by Russell (1980). Based on participant feedback during the pilot study, four additional words were added to the pool, making it 32 words. For visual stimuli, 30 one-lined black and white drawings were selected from Adobe Stock Library for which licenses were obtained, and the links to the pictures are provided in the Supplementary material. These 30 ambiguous images, where 15 images were used for each condition, were shuffled for every participant in all experimental and control conditions to control for order effects. A group of 10 working individuals, who were not a part of the actual experiment, comprising five females and five males within the age range of 26 to 46 years (mean = 36.0 years, SD = 1.41), were asked to rate the valence of these pictures on a scale of 1 = emotionally positive to 7 = emotionally negative. They rated most pictures as being emotionally neutral, with a mean rating of 3.73 (SD = 0.34) for 30 pictures.

To ensure reliability and applicability of the present research, a pilot study was conducted initially on five participants who were not a part of the final experiment. As a result, a consensus over the time limit of 1.5 min was agreed upon, and four additional words were added to the word pool for choosing three words to describe the ambiguous pictures in the final experiment. The pool of 32 words, shown in Table 1, was used to describe the ambiguous pictures in the final experiment.

**Table 1:** Pool of emotional words.

Pool of emotional words		
Sr. No.	English	Hindi
1.	Pleased	प्रसन्न
2.	Happy	खुश
3.	Sad	उदास
4.	Miserable	अत्यंत दुखी
5.	Calm	शांत
6.	Relaxed	आराम
7.	Serene	निर्मल / शांत
8.	Afraid	डरा हुआ
9.	Excited	उत्साहित
10.	Annoyed	चिड़चिड़ा
11.	Frustrated	हताश
12.	Tired	थका हुआ
13.	Delighted	अत्यंत प्रसन्न
14.	Content	संतुष्ट
15.	Distressed	पीड़ित
16.	Astonished	आश्चर्यचकित
17.	Angry	क्रोधित
18.	At ease	निश्चिंत
19.	Tense	तनावग्रस्त
20.	Shocked	चौंका हुआ
21.	Gloomy	मायूस
22.	Worried	चिंतित
23.	Lost	खोया हुआ
24.	Confused	भ्रमित
25.	Lonely	अकेला महसूस करना
26.	Hopeless	निराश
27.	Embarrassed	शर्मिंदा
28.	Overwhelmed	अभिभूत
29.	Inspired	प्रेरित
30.	Grateful	आभारी
31.	Bored	ऊबा हुआ
32.	Aroused	उत्तेजित

This table presents the pool of emotional words used in the emotional word selection task. For use in the Indian context, all words were translated into Hindi by a professional translator.

#### 2.3.3 Alternative Uses Task (AUT)

Initially proposed by Guilford in 1967, AUT is a well-validated tool that has undergone various refinements over the years (Guilford, 1967; Vartanian et al., 2020). This assessment provides an empirical measurement of creativity in order to capture the behavioral data. AUT operationalizes creativity, where the participants have to give as many unconventional potential uses of a random daily-use object (for instance, an umbrella, a paper clip) as possible. A growing body of studies has vouched for AUT over the years for being a reliable and valid tool for accurately measuring the creativity of the participants (Erwin et al., 2022). The total creativity score of AUT consists of three sub-dimensions: fluency, flexibility, and originality, rendering an overarching score of creativity. Fluency quantifies the number of ideas, flexibility evaluates the variety of response categories or class and originality, on the other hand, scores a point for the novelty of a given answer, in comparison to the other responses given by the participants. AUT has a notable experimental reliability and validity, with reliability (Cronbach’s *α*) results above 0.80 for fluency and higher (Benedek et al., 2013). Neuroscientific evidence via EEG, PET, and fMRI illustrated a coherence between AUT responses and the alpha activity of brain regions associated with creativity (Lloyd-Cox et al., 2022).

#### 2.3.4 Torrance Tests of Creative Thinking – Figural (TTCT-figural)

The Torrance Tests of Creative Thinking – Figural is a non-verbal, visual creativity assessment tool, which was developed by Ellis Paul Torrance in 1966. Torrance published the norms-technical manual, which provided expanded norms, reliability/validity data, and updated materials of TTCT-figural later (Torrance, 1974), and ever since, numerous researchers have propounded it as a well-validated tool and recognized it as a robust and reliable measure of creativity across cultures and age groups (Almeida et al., 2008; Kim, 2011). TTCT-figural operationalises creativity through visual and non-verbal divergent thinking tasks like picture construction, picture completion and the incomplete figures task, where the participants have to complete and transform the incomplete figures presented to them into meaningful and original drawings. TTCT-figural has these scoring metrics: fluency quantifies the number of ideas, flexibility highlights the variety of response categories or class, elaboration scores the detailing in the figure drawn, abstraction is the level of intellectual thinking for the title of the drawing and originality, on the other hand, measures the uniqueness or quality of a given answer in relation to the current pool of responses.

A large body of research has used TTCT-figural over the years; hence, endorsing its psychometric soundness, for instance, a recent reliability generalization meta-analysis reported composite reliability coefficients of 0.81 for overall scores, 0.85 for the innovative indices, and 0.62 for the adaptive paradigm (Acar, 2024). A specialized research study by Hahm et al. (2017) examined the brain functioning while performing Torrance Tests of Creative Thinking – Figural, found primary activation in the right fusiform gyrus, frontal, temporal, parietal, and occipital regions, along with widespread bilateral involvement. All these studies endorse TTCT-figural as a reliable tool for divergent figural creativity. The participants were given 2 minutes to complete and transform each incomplete figure into a meaningful and original drawing.

### 2.4 Procedure

The study employed a multifactorial design, with both within-subjects and between-subjects features. The experimental cohort adopted a within-subjects design, with participants undergoing both experimental conditions. In contrast, the control cohort used a between-subjects design, with participants undergoing the experimental task only once, without the music. Hence, the participants in the experimental cohort, with a two-day gap, underwent the experimental task twice, listening to two different ragas. In the preliminary stages preceding the formal experiment, participants completed an online google form with their demographic details relevant to the study’s inclusion and exclusion criteria. Then all the participants completed an online Positive Affect and Negative Affect Schedule (PANAS) to assess their mood and emotional valence over the past week. The participants who met the inclusion and exclusion criteria were randomly assigned to either the experimental or control cohort and subsequently invited to the research site to complete the formal experiment under the supervision of the researcher.

After arriving at the research site, the investigator informed the participants about the aims and objectives of the study; thereafter, written consent from the participants was obtained. Before commencing the main experiment, participants were given three practice questions of each task to ensure familiarity with the tasks. The experimental task was conducted individually, such that only one participant was tested at a time. The duration of the task ranged from 45 min for the experimental cohort, as they had to hear the musical piece as well and 30 min for the control cohort. The participants attempted the experimental task on an iPad Air (5th generation), provided to them by the researcher.

The experimental task was presented via Jotform, an online forms platform. Then they were provided a pair of headphones (Zebronics Thunder Bluetooth headphones) to hear the raga for 12 min. Thereafter, participants proceeded to the Emotional Word Selection Task, where they were shown an ambiguous image and for each image they had to choose three emotional words out of a pool of 32 words to describe the image. A total of 15 pictures were presented in a single experimental task. There was a time limit of 90 s for each picture (decided based on the pilot study), where they also rated the intensity of the three words chosen for each image on a 5-point Likert scale. Minimal time was given for the task to catch the very first instinctual perception of the image. The Emotional Word Selection Task ran smoothly on the JotForm, where the images were presented on their own with a timer, along with the words to select and then to rate the intensity of the selected words.

Immediately after, instructions for the next task, which was the Alternative Uses Task, were displayed on the screen. Since the participants were already acquainted with all the tasks, they straight away progressed to the next task. Participants were given a random daily-use object (for instance, an umbrella, a paper clip) and they had to type in the space given on the screen as many unconventional potential uses of the object as possible, within 2 minutes for each object. A total of two objects were given in the task.

Then, instructions for the next task, which was the Torrance Tests of Creative Thinking – Figural, were displayed on the screen. Participants were already acquainted with the task and how to draw using an apple pencil on an iPad. Post instructions, an incomplete figure was displayed on the screen. Participants had to complete this incomplete figure and draw a meaningful figure or drawing from it, and then give an appropriate title to it. Two incomplete figures were given in the task, with a time limit of 2 minutes per figure.

**Figure 1:**
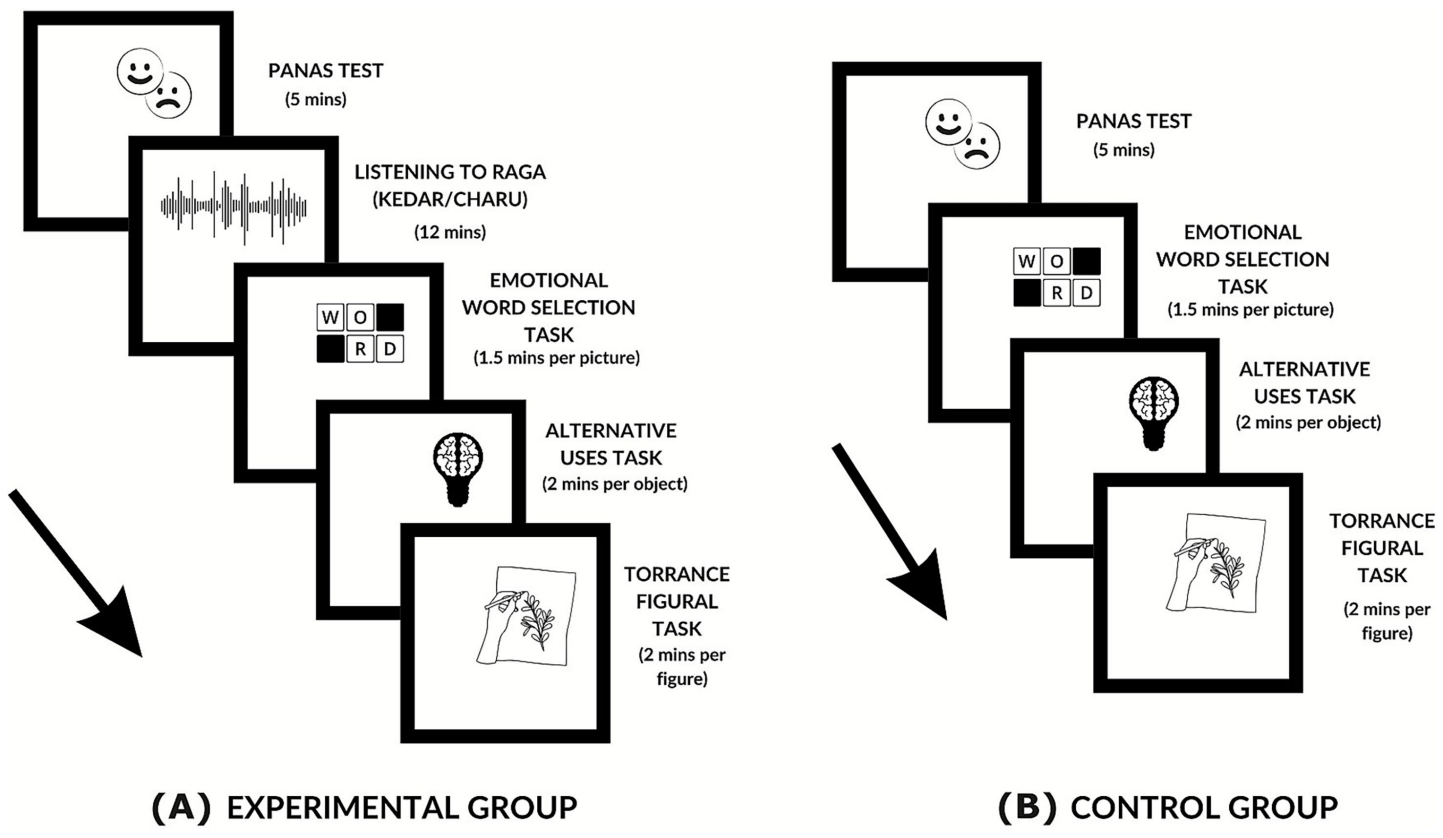
Sequence of experimental tasks. **(A)** The experimental task of the experimental group progressed in four sequential stages: (1) Listening to a raga (12 min), (2) Emotional Word Selection Task for describing ambiguous images (1.5 min per picture; 15 pictures), (3) Alternative Uses Task (two mins per object; two objects), (4) Torrance Tests of Creative Thinking – Figural (two mins per figure; two figures). **(B)** The experimental task of the control group excluded exposure to the raga in the sequence. Figure created by the author using Canva (https://www.canva.com/), and for exporting the figure in high resolution, Inkscape (https://inkscape.org/) software was used. Icons used from Canva’s licensed library.

After completing all the tasks, participants were asked a few questions regarding their thoughts on the emotional valence of the music, whether they liked it or not, whether the music had changed their mood or evoked any emotions, and whether the music had an impact on their perception or creativity. The experimental procedure of the control group was identical to that of the experimental group, with the exception of not being exposed to any priming music. All other aspects of task order, timing, and instructions for the Emotional Word Selection Task and creativity measures were constant across groups. The absence of an auditory stimulus served as the no-prime baseline for the between-group comparisons. The sequential procedure of the experimental and the control group is illustrated in Figure 1.

Link of experimental task: https://form.jotform.com/250785755030458.

Link to the 2nd part of the experimental task: https://form.jotform.com/250784595806066.

### 2.5 Data design and analysis

The statistical software IBM SPSS Statistics was used for the data analyses in the study (IBM Corp, 2023). The research design employed by the study was a multifactorial design, with both within-subjects and between-subjects features. A repeated-measures ANOVA and a multivariate ANOVA were conducted to assess the data. Participants completed the Emotional Word Selection Task and two creativity tests — the Alternative Uses Task and the Torrance Tests of Creative Thinking - Figural after listening to each raga or no raga. For each measure, three comparisons were conducted. Firstly, the difference between the experimental conditions (Raga Kedar and Raga Charukeshi) was evaluated using repeated-measures ANOVA, as the participants of the experimental group were exposed to both conditions (within-subject differences). Secondly, the difference between each experimental condition and the control group (no raga) was evaluated separately for both experimental conditions using multivariate analyses of variance (MANOVA) as the experimental and control group involved separate participants (between-subject differences). All the analyses included differences based on age group and gender as between-subjects factors.

Age group 25–35:- 30 participants in total = 15 in experimental; 15 in control group.

Age group 36–45:- 30 participants in total = 15 in experimental; 15 in control group.

Age group 46–55:- 30 participants in total = 15 in experimental; 15 in control group.

## 3 Results

### 3.1 Mood assessment and its predictive role in emotional word choice

Linear regression analyses were conducted to examine whether the mood of the participants, as measured by the Positive Affect and Negative Affect Schedule (PANAS), predicted the emotional word choices in the Emotional Word Selection Task of the participants to describe the ambiguous images, across experimental conditions (Kedar, Charukeshi, and control), as shown in Table 2.

**Table 2:** Linear regression analysis predicting emotional word choices (positive and negative) from PANAS scores across experimental conditions (Kedar, Charukeshi, and Control), which shows that the mood of the participants did not reliably predict the emotional word choices of the participants in the emotional Word Selection Task to describe the ambiguous images in any of the experimental or control conditions.

Group	Dependent variable	Predictor	*B*	SE	*t*	*p*	*R* ^2^
Kedar	Positive words choice	Positive affect	0.934	0.551	1.70	0.097	0.063
	Negative words choice	Negative affect	−0.528	0.399	−1.32	0.193	0.039
Charukeshi	Positive words choice	Positive affect	0.941	0.558	1.69	0.099	0.062
	Negative words choice	Negative affect	0.862	0.446	1.93	0.060	0.080
Control	Positive words choice	Positive affect	1.050	0.844	1.24	0.222	0.035
	Negative words choice	Negative affect	– 0.716	0.551	−1.30	0.201	0.038

The positive affect score of the Kedar condition, did not significantly predict positive word choices of the participants [*B* = 0.934, SE = 0.551, *t*(43) = 1.70, *p* = 0.097, *R*^2^ = 0.063], and negative affect score did not significantly predict negative word choices of the participants [*B* = −0.528, SE = 0.399, *t*(43) = −1.32, *p* = 0.193, *R*^2^ = 0.039].

Similarly, the positive affect score of the Charukeshi condition, did not significantly predict positive word choices of the participants [*B* = 0.941, SE = 0.558, *t*(43) = 1.69, *p* = 0.099, *R*^2^ = 0.062], and negative affect score did not significantly predict negative word choices of the participants [*B* = 0.862, SE = 0.446, *t*(43) = 1.93, *p* = 0.060, *R*^2^ = 0.080].

For the control group, the positive affect score did not significantly predict positive word choices of the participants [*B* = 1.050, SE = 0.844, *t*(43) = 1.24, *p* = 0.222, *R*^2^ = 0.035], and negative affect score did not significantly predict negative word choices of the participants [*B* = − 0.716, SE = 0.551, *t*(43) = −1.30, *p* = 0.201, *R*^2^ = 0.038].

Across all three conditions, the results indicate that the mood of the participants, as measured by PANAS, did not reliably predict the emotional word choices in the Emotional Word Selection Task of the participants to describe the ambiguous images in any of the experimental or control conditions.

### 3.2 Affective priming effects on emotional word selection

**Figure 2:**
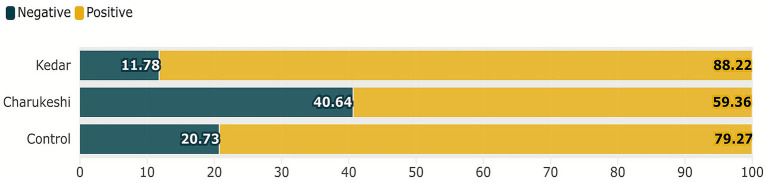
Mean percentage differences in the Emotional Word Selection Task. The figure presents the mean percentage of positive and negative words chosen after exposure to Raga Kedar, Raga Charukeshi, and the control condition (no raga). The highest percentage of positive words was chosen after exposure to Raga Kedar (88.22%) compared to Raga Charukeshi (59.36%) and the control group (79.27%). Conversely, the highest percentage of negative words was chosen after exposure to Raga Charukeshi (40.64%) compared to Raga Kedar (11.78%) and the control group (20.73%), with the control group defaulting to a near-neutral baseline. Bar chart created by the author using Flourish (https://flourish.studio), and for exporting the figure in high resolution, Inkscape (https://inkscape.org/) software was used.

**Figure 3:**
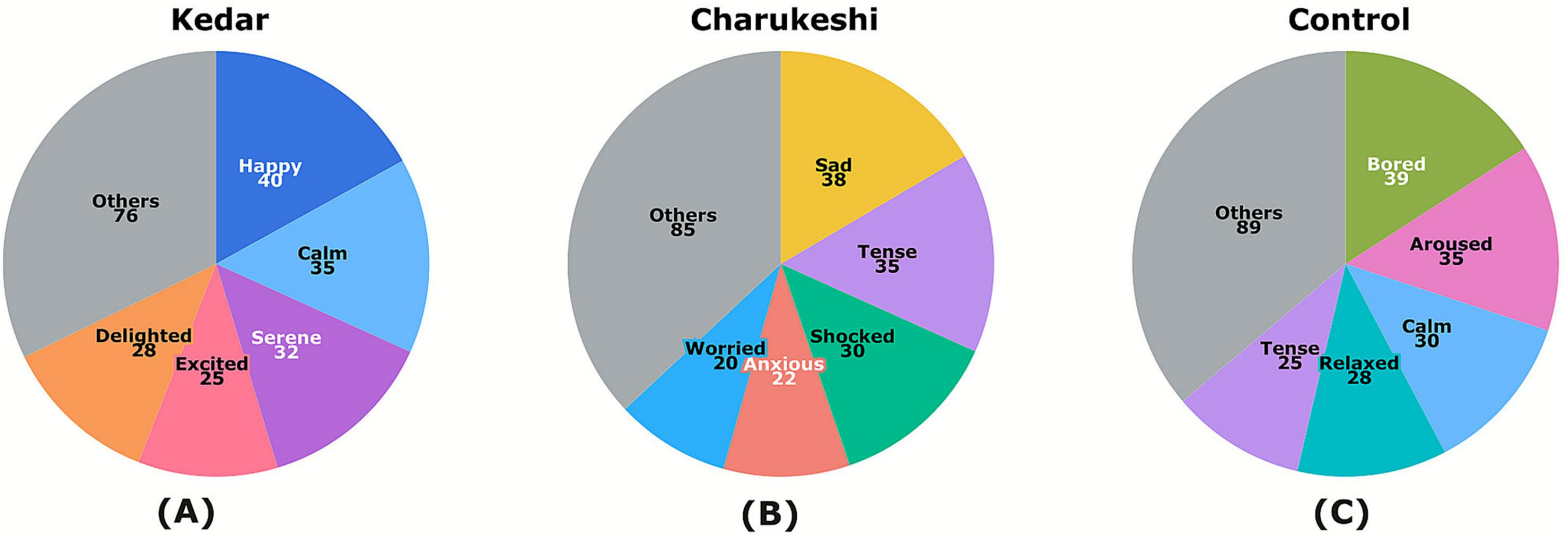
Distribution of emotional words selected in the Emotional Word Selection Task across **(A)** Kedar, **(B)** Charukeshi, and **(C)** control conditions. The pie charts illustrate the percentage distribution of the most commonly chosen words for each condition, with the other responses grouped into the ‘Other entries’ category. Pie charts created by the author using Flourish (https://flourish.studio), and for exporting the figure in high resolution, Inkscape (https://inkscape.org/) software was used.

In the Emotional Word Selection Task, as illustrated in Figure 2, after exposure to Raga Kedar, participants chose the highest number of positive words (*M* = 147.76, SD = 30.11), followed by the control condition (*M* = 128.62, SD = 38.70) and the least number of positive words after exposure to Raga Charukeshi (*M* = 81.67, SD = 29.51). Conversely, after exposure to Raga Kedar, participants chose the least number of negative words (*M* = 19.73, SD = 16.62), followed by the control condition (*M* = 33.64, SD = 22.61) and the highest number of negative words after exposure to Raga Charukeshi (*M* = 55.91, SD = 22.46). The percentage distribution of the most commonly chosen words for each condition is illustrated in Figure 3.

#### 3.2.1 Raga Kedar vs. Raga Charukeshi (repeated-measures ANOVA)

Before conducting a repeated-measures ANOVA to analyze the difference between the experimental conditions (Raga Kedar and Raga Charukeshi), assumption checks were performed. Mauchly’s test of sphericity indicated that for the ragas x valence factor, sphericity was violated *χ*^2^(2) = 33.313, *p* < 0.001. Hence, Greenhouse–Geisser correction was applied to the within-subject effects.

Pillai’s Trace indicated a significant effect of raga, *V* = 0.385, *F*(1,39) = 24.368, *p* < 0.001, ηp^2^ = 0.385; and for ragas x valence interaction, *V* = 0.768, *F*(2,38) = 62.725, *p* < 0.001, ηp2 = 0.768 which indicates that the pattern of emotional word use across valence levels differed depending on the raga. These results were followed by univariate tests, which confirmed that these effects were consistent across the individual dependent variables. The pairwise comparisons of the estimated marginal means indicated that participants chose more positive words after listening to Raga Kedar than Raga Charukeshi (mean difference = 66.057, *p* < 0.001, 95% CI [53.79, 78.31]), whereas they chose more negative words after listening to Raga Charukeshi than Raga Kedar (mean difference = 36.429, *p* < 0.001, 95% CI [28.18, 44.67]).

#### 3.2.2 Raga Kedar vs. control group (MANOVA)

Before conducting MANOVA to analyze the difference between Raga Kedar and the control group, assumption checks were performed. The assumption of equality of covariance matrices was met, as indicated by Box’s *M* test, *M* = 86.165, *p* = 0.383. The assumption of homogeneity of variances was met for all dependent variables, as indicated by Levene’s test based on mean for positive words, *F*(11,78) = 1.789, *p* = 0.070 and negative words, *F*(11,78) = 0.956, *p* = 0.493.

Pillai’s Trace indicated a significant multivariate effect of group, *V* = 0.144, *F*(3,76) = 4.25, *p* = 0.008, ηp^2^ = 0.144. These results were followed by univariate tests, which confirmed that these effects were consistent across the individual dependent variables. The pairwise comparisons of the estimated marginal means indicated that participants chose more positive words after listening to Raga Kedar than control group (mean difference = 21.104, *p* = 0.004, 95% CI [7.03, 35.17]), whereas, conversely they chose slightly more negative words without any raga in comparison to Raga Kedar (mean difference = 13.792, *p* < 0.001, 95% CI [5.48, 22.09]).

#### 3.2.3 Raga Charukeshi vs. control group (MANOVA)

Before conducting MANOVA to analyze the difference between Raga Charukeshi and the control group, assumption checks were performed. The assumption of equality of covariance matrices was met, as indicated by Box’s *M* test, *M* = 82.937, *p* = 0.443. The assumption of homogeneity of variances was met for all dependent variables, as indicated by Levene’s test based on mean for positive words *F*(11,78) = 1.439, *p* = 0.173 and negative words *F*(11,78) = 0.400, *p* = 0.952.

Pillai’s Trace indicated a significant multivariate effect of group, *V* = 0.345, *F*(3,76) = 13.365, *p* < 0.001, ηp^2^ = 0.345. These results were followed by univariate tests, which confirmed that these effects were consistent across the individual dependent variables. The pairwise comparisons of the estimated marginal means indicated that participants chose more positive words without listening to any raga (control group) in comparison to Raga Charukeshi (mean difference = 47.042, *p* < 0.001, 95% CI [32.10, 61.97]), whereas, conversely they chose more negative words after listening to Raga Charukeshi than control group (mean difference = 21.82, *p* < 0.001, 95% CI [12.24, 31.40]).

### 3.3 Affective priming effects on divergent thinking

**Figure 4:**
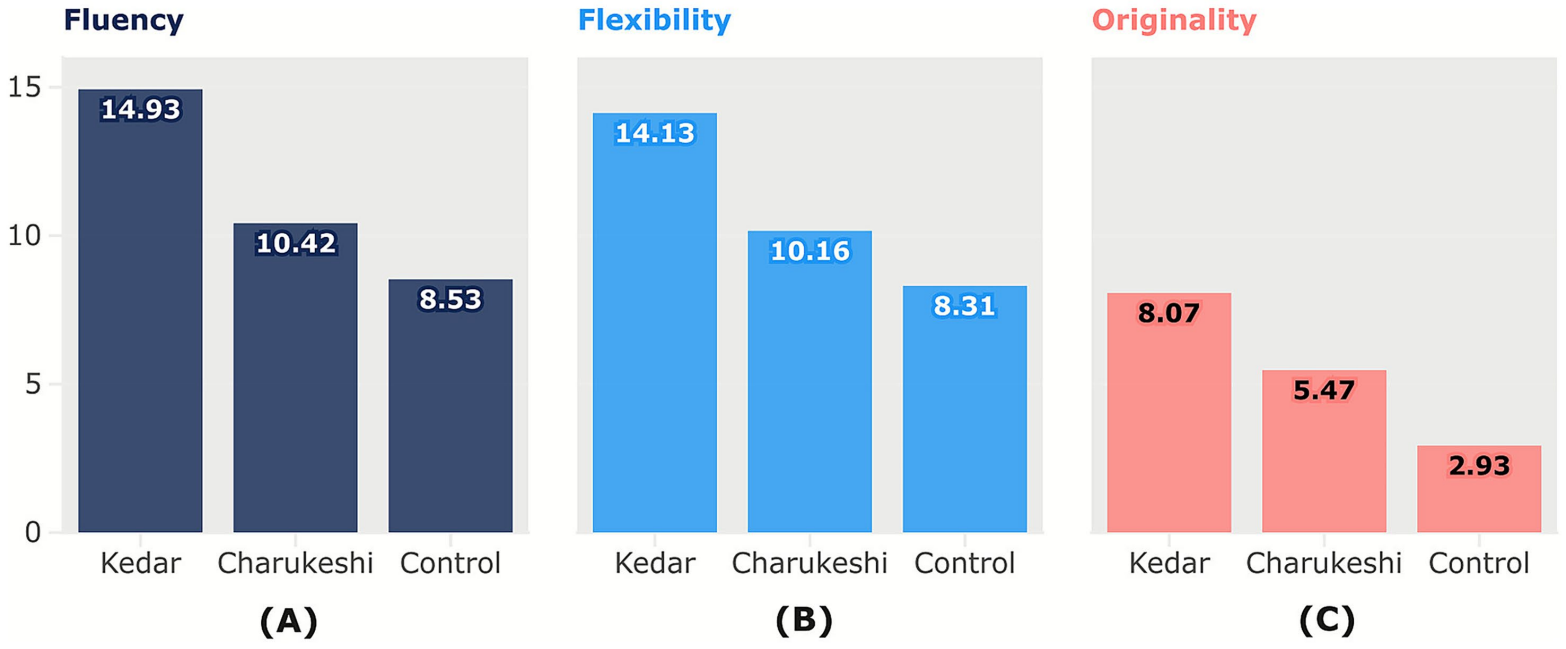
Mean differences in the Alternative Uses Task. The figure presents the mean differences in performance on **(A)** fluency, **(B)** flexibility, and **(C)** originality (subtests of AUT) after exposure to Raga Kedar, Raga Charukeshi, and the control condition (no raga) in AUT. Raga Kedar consistently resulted in the highest creativity scores on all creativity subtests, while Raga Charukeshi outperformed the control group on all creativity subtests, though scored less than Raga Kedar. This indicates that although both ragas increased creativity, the degree of impact depends on the emotional valence of the music. Column chart created by the author using Flourish (https://flourish.studio), and for exporting the figure in high resolution, Inkscape (https://inkscape.org/) software was used.

**Figure 5:**
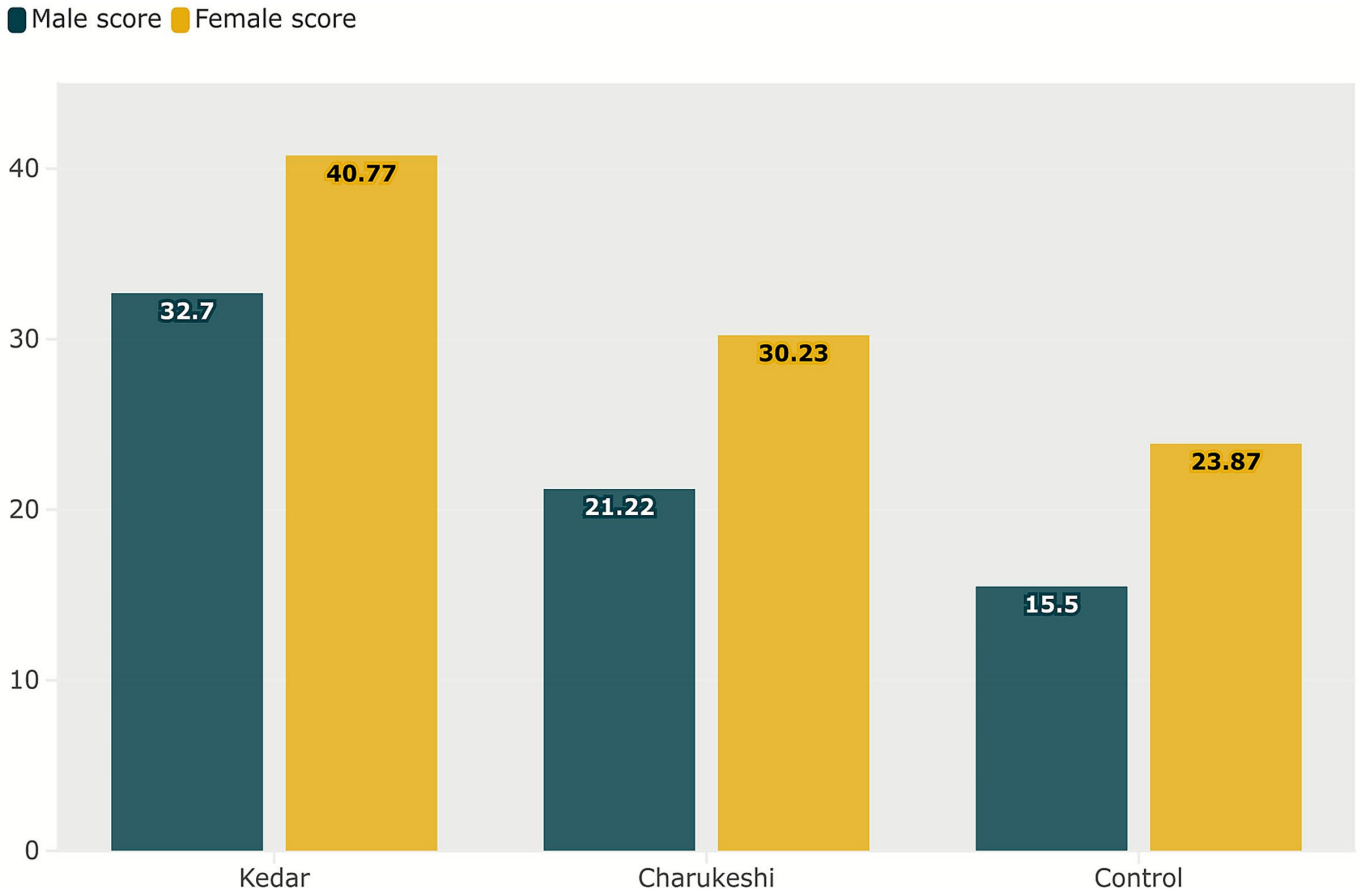
Mean gender differences in the Alternative Uses Task. The figure presents the mean differences in the overall performance of males and females on AUT, which reveals that females outperformed the male participants, scoring higher in fluency, flexibility, and originality. Column chart created by the author using Flourish (https://flourish.studio), and for exporting the figure in high resolution, Inkscape (https://inkscape.org/) software was used.

As illustrated in Figure 4, the creativity of participants in the Alternative Uses Task (AUT) based on total score was highest after exposure to Raga Kedar (*M* = 36.64, SD = 9.580), followed by Raga Charukeshi (*M* = 25.62, SD = 9.79) and was the lowest without any raga (*M* = 19.78, SD = 8.62). The mean gender differences in the overall performance are illustrated in Figure 5.

#### 3.3.1 Raga Kedar vs. Raga Charukeshi (repeated-measures ANOVA)

Before conducting a repeated-measures ANOVA to analyze the difference between the experimental conditions (Raga Kedar and Raga Charukeshi), assumption checks were performed. Mauchly’s test of sphericity indicated that for the creativity factor, sphericity was violated *χ*^2^(2) = 85.226, *p* < 0.001, and for ragas x creativity factor, sphericity was violated *χ*^2^(2) = 78.843, *p* < 0.001. Hence, Greenhouse–Geisser correction was applied to the within-subject effects. The assumption of homogeneity of variances was met, as indicated by Levene’s tests, for all dependent variables (*p*s > 0.05).

Pillai’s Trace indicated a significant effect of raga, *V* = 0.654, *F*(1,39) = 73.749, *p* < 0.001, ηp^2^ = 0.654; and of ragas x creativity interaction, *V* = 0.163, *F*(2,38) = 3.697, *p* = 0.034, ηp^2^ = 0.163. These results were followed by univariate tests, which confirmed that these effects were consistent across the individual dependent variables. The between-subjects effect of gender was significant, *F*(1,43) = 15.88, *p* < 0.001, ηp^2^ = 0.289. The pairwise comparisons of the estimated marginal means indicated that participants’ fluency (mean difference = 4.49, *p* < 0.001), 95% CI [3.17, 5.82], flexibility (mean difference = 3.98, *p* < 0.001), 95% CI [2.89, 5.07], and originality (mean difference = 2.65, *p* < 0.001), 95% CI [1.56, 3.74], increased after listening to Raga Kedar in comparison to Raga Charukeshi. Pairwise comparison of gender indicated that female participants scored higher overall on the AUT compared to male participants (mean difference = 3.033, *p* < 0.001), 95% CI [1.49, 4.57].

#### 3.3.2 Raga Kedar vs. control group (MANOVA)

Before conducting MANOVA to analyze the differences between Raga Kedar and the control group, assumption checks were performed. The assumption of equality of covariance matrices, as indicated by Box’s *M* test, was not met, *M* = 127.113, *p* = 0.003; hence, Pillai’s Trace was used for the multivariate tests. Levene’s test indicated violations of the homogeneity of variance assumption for all dependent variables, so univariate between-subjects effects were not interpreted.

Pillai’s Trace indicated a significant multivariate effect of group [*V* = 0.588, *F*(3,76) = 36.150, *p* < 0.001, ηp^2^ = 0.588] and gender [*V* = 0.246, *F*(3,76) = 8.250, *p* < 0.001, ηp^2^ = 0.246]. The pairwise comparisons of the estimated marginal means indicated that participants’ fluency (mean difference = 6.40, *p* < 0.001), 95% CI [5.11, 7.69], flexibility (mean difference = 5.83, *p* < 0.001), 95% CI [4.64, 7.03], and originality (mean difference = 5.23, *p* < 0.001), 95% CI [3.76, 6.70], increased after listening to Raga Kedar in comparison to the control group. Pairwise comparison of gender indicated that female participants scored higher overall on the AUT in fluency (mean difference = 2.66, *p* < 0.001), 95% CI [1.36, 3.95], flexibility (mean difference = 2.46, *p* < 0.001), 95% CI [1.26, 3.66] and originality (mean difference = 3.12, *p* < 0.001), 95% CI [1.65, 4.58] compared to male participants.

#### 3.3.3 Raga Charukeshi vs. control group (MANOVA)

Before conducting MANOVA to analyze the difference between Raga Charukeshi and the control group, assumption checks were performed. The assumption of equality of covariance matrices, as indicated by Box’s *M* test, was not met, *M* = 136.153, *p* < 0.001; hence, Pillai’s Trace was used for the multivariate tests. Levene’s test indicated violations of the homogeneity of variance assumption for all dependent variables, so univariate between-subjects effects were not interpreted.

Pillai’s Trace indicated a significant multivariate effect of group [*V* = 0.179, *F*(3,76) = 5.53, *p* = 0.002, ηp^2^ = 0.179] and gender [*V* = 0.262, *F*(3,76) = 8.98, *p* < 0.001, ηp^2^ = 0.262]. The pairwise comparisons of the estimated marginal means indicated that participants’ fluency (mean difference = 1.90, *p* = 0.007, 95% CI [0.53, 3.28]), flexibility (mean difference = 1.85, *p* = 0.006, 95% CI [0.54, 3.16]), and originality (mean difference = 2.57, *p* < 0.001, 95% CI [1.32, 3.82]), increased after listening to Raga Charukeshi than listening to no raga. Pairwise comparison of gender indicated that female participants scored higher overall on the AUT in fluency (mean difference = 3.11, *p* < 0.001, 95% CI [1.74, 4.48], flexibility (mean difference = 2.92, *p* < 0.001, 95% CI [1.62, 4.23] and originality (mean difference = 3.018, *p* < 0.001, 95% CI [1.76, 4.26] compared to male participants.

### 3.4 Affective priming effects on figural creativity

**Figure 6:**
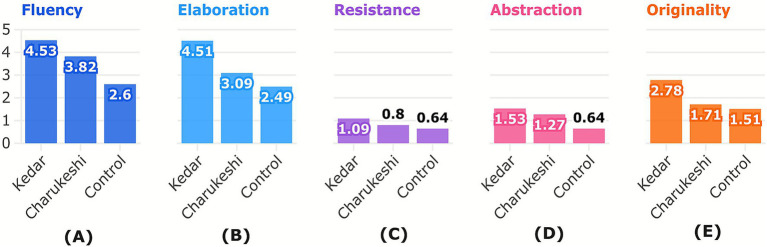
Mean differences in the Torrance Tests of Creative Thinking – Figural. The figure presents the mean differences in performance on **(A)** Fluency, **(B)** elaboration, **(C)** resistance, **(D)** abstraction, and **(E)** originality (subtests of TTCT-Figural) after exposure to Raga Kedar, Raga Charukeshi, and the control condition (no raga) in TTCT-Figural. Raga Kedar consistently yielded the highest creativity scores on all creativity subtests, while Raga Charukeshi outperformed the control group on all subtests, though scoring lower than Raga Kedar. This indicates that although both ragas increase creativity, the degree of impact depends on the emotional valence of the music. Column chart created by the author using Flourish (https://flourish.studio), and for exporting the figure in high resolution, Inkscape (https://inkscape.org/) software was used.

**Figure 7:**
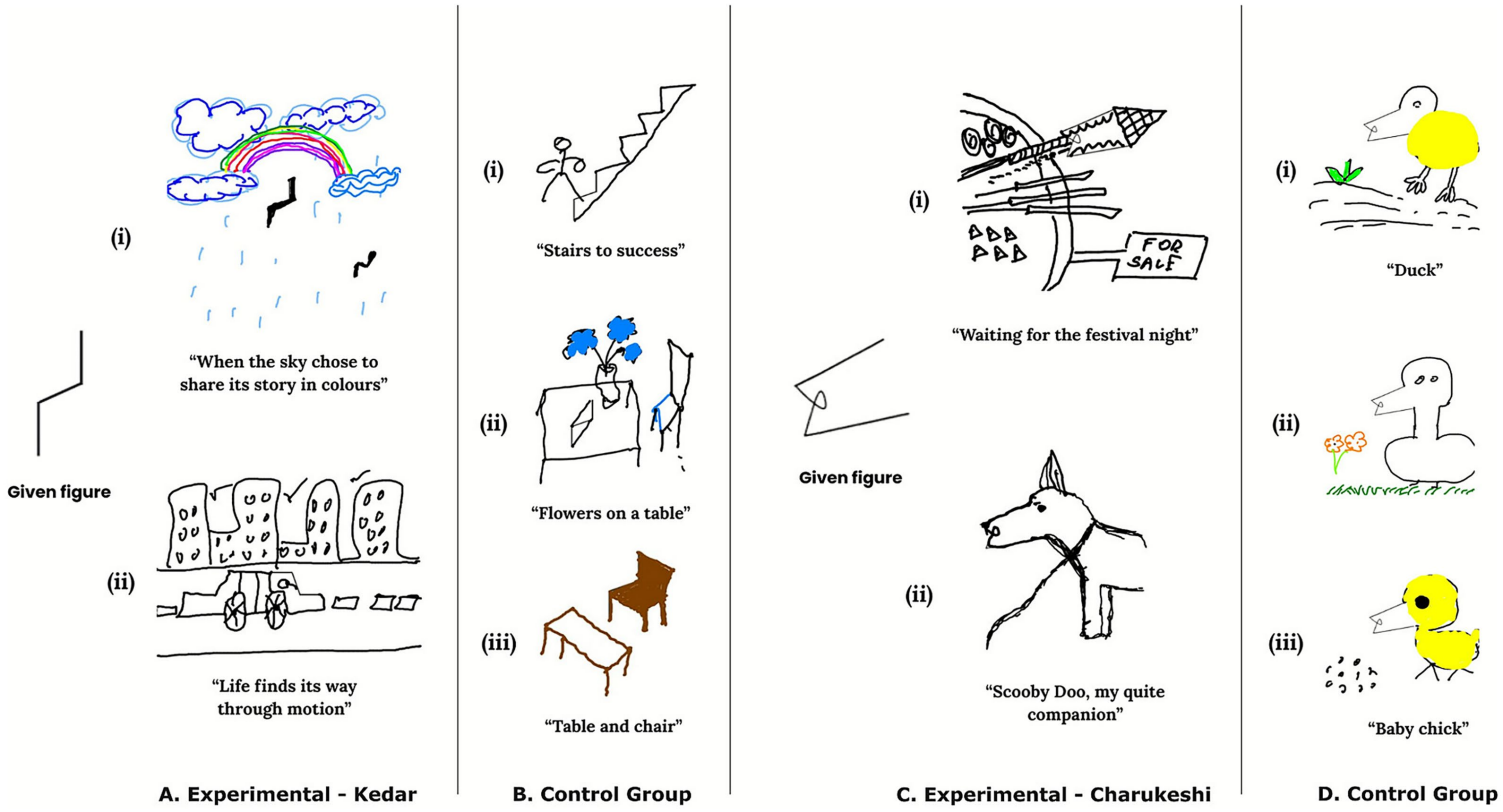
Representative drawings from the Torrance Tests of Creative Thinking – figural across **(A)** Experimental - Kedar, **(B)** control group, **(C)** Experimental - Charukeshi, and **(D)** control group. **(A,C)** Depict responses of the experimental group on the TTCT figural after exposure to Raga Kedar and Raga Charukeshi, respectively. **(B,D)** Depict the responses of the control group, who completed the task only once without exposure to any music; these panels depict their performance of two given figural stimuli in a single experimental session. Figure created by the author using Canva (https://www.canva.com/) to combine all the figural drawings, and for exporting the figure in high resolution, Inkscape (https://inkscape.org/) software was used.

As illustrated in Figure 6, the creativity of participants in Torrance Tests of Creative Thinking - Figural was highest after exposure to Raga Kedar (*M* = 14.44, SD = 5.62), followed by Raga Charukeshi (*M* = 10.16, SD = 4.27) and was the lowest without any raga (*M* = 7.76, SD = 3.04). The differences in the performance of the experimental and control group on the TTCT-figural can be seen in Figure 7.

#### 3.4.1 Raga Kedar vs. Raga Charukeshi (repeated-measures ANOVA)

Before conducting a repeated-measures ANOVA to analyze the difference between the experimental conditions (Raga Kedar and Raga Charukeshi), assumption checks were performed. Mauchly’s test of sphericity indicated that for the creativity factor, sphericity was violated χ^2^(2) = 21.82, *p* = 0.010, and for ragas x creativity factor as well, sphericity was violated χ^2^(2) = 25.590, *p* = 0.002. Hence, Greenhouse–Geisser correction was applied to the within-subject effects.

Pillai’s Trace indicated a significant effect of raga, *V* = 0.589, *F*(1,39) = 55.924, *p* < 0.001, ηp^2^ = 0.589; of creativity, *V* = 0.828, *F*(4,36) = 43.222, *p* < 0.001, ηp^2^ = 0.828 and of ragas x creativity interaction, *V* = 0.243, *F*(4,36) = 2.883, *p* = 0.036, ηp^2^ = 0.243. These results were followed by univariate tests, which confirmed that these effects were consistent across the individual dependent variables. The pairwise comparisons of the estimated marginal means indicated that participants scored higher on AUT after listening to Raga Kedar than Raga Charukeshi (mean difference = 0.79, *p* < 0.001, 95% CI [0.58, 1.01]).

#### 3.4.2 Raga Kedar vs. control group (MANOVA)

Before conducting MANOVA to analyze the difference between Raga Kedar and the control group, assumption checks were performed. The assumption of equality of covariance matrices, as indicated by Box’s *M* test, was not met, *M* = 314.87, *p* = 0.011; hence, Pillai’s Trace was used for the multivariate tests. The assumption of homogeneity of variances was met, as indicated by Levene’s tests, for all dependent variables (*p*s > 0.05).

Pillai’s Trace indicated a significant multivariate effect of group [*V* = 0.371, *F*(5,74) = 8.722, *p* < 0.001, ηp^2^ = 0.371]. The pairwise comparisons of the estimated marginal means indicated that participants’ fluency (mean difference = 1.90, *p* < 0.001, 95% CI [1.09, 2.72]), elaboration (mean difference = 2.06, *p* < 0.001, 95% CI [1.24, 2.88]), resistance (mean difference = 0.45, *p* = 0.025, 95% CI [0.05, 0.84]), abstraction (mean difference = 0.86, *p* = 0.005, 95% CI [0.27, 1.46]), and originality (mean difference = 1.26, *p* = 0.001, 95% CI [0.51, 2.01]) increased after listening to Raga Kedar in comparison to the control group.

#### 3.4.3 Raga Charukeshi vs. control group (MANOVA)

Before conducting MANOVA to analyze the difference between Raga Charukeshi and the control group, assumption checks were performed. The assumption of equality of covariance matrices, as indicated by Box’s *M* test, was not met, *M* = 309.895, *p* = 0.016; hence, Pillai’s Trace was used for the multivariate tests. Pillai’s Trace indicated a significant multivariate effect of group [*V* = 0.173, *F*(5,74) = 3.088, *p* = 0.014, ηp^2^ = 0.173].

## 4 Discussion

The principal objective of this investigation was to probe the effects of affective priming through Indian ragas on the perception of ambiguous visual stimuli and creativity, which evinces a discernible unconscious influence of affective priming through music on subsequent behavior. The results of a large-scale project with cross-cultural evidence, analyzing nearly one million listener ratings of songs from 59 countries, concluded that the valence-arousal structure of emotional response to music is consistently shared across cultures (Lee et al., 2025). This alludes that in spite of unfamiliarity with Indian ragas, it can unconsciously express congruent emotional valence, yielding an impact on perception and enhancement of creativity.

### 4.1 Limited influence of baseline mood on emotional word choice

Linear regression analyses were conducted to examine whether the mood of the participants, as measured by the PANAS, predicted the emotional word choices in the Emotional Word Selection Task, which participants used to describe ambiguous images across experimental conditions (Kedar, Charukeshi, and control). Across all three conditions, the results indicated that the mood of the participants, as measured by PANAS, did not predict the emotional word choices of the participants to describe the ambiguous images in any of the experimental or control conditions. These findings suggest that the changes in the lexical choices were not driven by the prior affective states of the participants and are more plausibly attributable to the affective priming through exposure to ragas, which substantiates the findings of this research.

### 4.2 Top-down influence of priming on perception of ambiguous visual stimuli

The study hypothesized that ragas as affective primes would serve as a critical determinant in shaping the perception of the ambiguous images, thereby imprinting congruent semantic lexical choice with the affect of the music for describing them in the Emotional Word Selection Task. Assuming that participants exposed to Raga Kedar (positive raga) would choose more words of positive tenor (happy, calm, relaxed), those exposed to Raga Charukeshi, which was of negative composition, would choose more words of negative tenor (miserable, sad, angry), while the control group would default to neutrality (bored, lost). The results expound a notable difference between each experimental condition and the control group. Relative to the control condition, participants chose more positive words after listening to Raga Kedar and fewer negative words. Conversely, after listening to Raga Charukeshi, they chose more negative words and fewer positive words to describe the ambiguous visual images. The control group defaulted to neutrality. This illustrates that positive music unconsciously primed the participants’ perception of the ambiguous visual stimuli, leading them to choose more positive words. Conversely, the same participants chose more negative responses for ambiguous visual images after listening to Raga Charukeshi (sad music).

Preceding studies enumerate that beyond basic emotions, music conveys affect (Cespedes-Guevara and Eerola, 2018), and it acts as the “language of emotions” (Juslin, 2013). In a similar vein, the ragas in this study disseminate a specific affective tone, which was consistent with their emotional associations. Drawing upon neuroimaging evidence using fMRI, Brattico et al. (2011) illustrated that by listening to happy and sad music with and without lyrics, brain regions involved in affective and reward-related processing are activated, contending that music reliably conveys emotional meaning at the neural level, which buttresses this standpoint.

Event-related potentials (ERPs) have been operationalized to testify that music instantiates change in the semantic processing of words via affective priming by displaying that incongruence between the musical prime and the target word elicited stronger N400 responses, which denotes that meaning processing at the neural level can be explicitly modulated by music (Steinbeis and Koelsch, 2011). Within the realm of music, another language-based study was conducted by Tay and Ng (2019), who employed lexical choice to demonstrate that affective priming through music dictated the participants’ linguistic choices, where the emotional words selected were congruent with the valence of the music. Huziwara et al. (2022) also yielded discernible evidence in favor that consonant (happy) and dissonant (sad) musical chords expedited facial expression recognition for congruent facial emotions. The assembled findings foreground the results of the present study, where ragas acted as affective primes that guided participants’ emotional word use in a congruent direction.

It has been discovered by Pottratz et al. (2020) that even though participants were not consciously attending to the prime, subliminal primes embedded in music videos influenced them with positively valenced affect, illustrating the unconscious influence of priming. Analogously, Li et al. (2023) adduced empirical fMRI data attesting that when participants imagined emotional scenes while listening to emotionally congruent music, their neural activation in the subcortical and limbic areas of the brain, such as the hypothalamus, brainstem, amygdala, and hippocampus, was more pronounced compared to imagery without music, showcasing in-depth neural engagement via music. This plausibly suggests that music-evoked affect by ragas in the present study may likewise have triggered unconscious affective neural processes.

In the present study, the same ambiguous images were interpreted differently by the participants of the experimental group and the control group, who chose different emotional words that were congruent with the emotional state of the raga. Although the visual stimuli remained unchanged, different words were chosen, pointing toward the inference that the affective states shape our perceptual experience. Hence, affective priming does more than just tweak what words people choose; it indeed changes how people perceive the ambiguous stimuli. Their response patterns reflect a prior expectation or affective bias, challenging the purely stimulus-driven Bottom-up processing, henceforth signaling that their perception was biased. The findings of this research elucidate robust evidence for the top-down model of perceptual processing, stating that the perceptual experience was an amalgamation of the emotional state influenced by the ragas and the external stimuli. This interpretation is consistent with Jolij and Meurs (2011), who demonstrated that affective priming provides compelling evidence for Top-Down Processing, explicating that mood induced via music biases visual perception.

While ragas in the present study were found to be robust affective primes for lexical choice, it is imperative to assess these results against the background of broader literature, which reported null or tenuous priming effects. No priming effect was observed by Goerlich et al. (2012) when the valence of the prime was not congruent with the task (classify words according to their size or color, instead of emotions) and Armitage and Eerola (2022) outlined that the priming effect was observed only in a valence task but not in a lexical decision task, which reveals that priming is not fully automatic but rather task-specific to the emotional dimension instead of being generalizable across tasks. These findings are not a direct refutation of the present study, as it employed a sustained, consciously perceived musical stimulus (i.e., extended, affectively rich, and temporally unfolding), but rather a cautionary backdrop regarding the methodological and contextual boundary conditions. While the study incorporated a supraliminal priming framework in which the consciously perceived stimuli exert a downward implicit influence, the extended conscious exposure to the stimuli also introduces the possibility of expectancy and demand-related effects; hence, the findings should be interpreted cautiously.

In comparison to the control group, Raga Charukeshi exhibited a larger effect size (np^2^ = 0.345) than Raga Kedar (np^2^ = 0.144), indicating that exposure to Raga Charukeshi had a stronger influence on participants’ word selection for ambiguous images. This asymmetry is well documented in these classic studies by Baumeister et al. (2001) as well as Rozin and Royzman (2001), who highlighted the concept of ‘negativity bias’, illustrating that negative affect exerts a stronger and more persistent influence on our cognition and perception than positive affect. Notably, Norris (2019) reviewed multiple ERP and fMRI studies that negative information receives more neural processing, underscoring our findings that negative affect primed through Raga Charukeshi had a more powerful and persistent effect than positive affect. There were no significant gender or age group differences found in the study, which makes these results robust and generalizable across both males and females of the 25–55 age group. These results can be effectively applied to educational, therapeutic and workplace settings as carefully selected music can help in priming positive emotional framing, regulating emotions, cognitive-emotional training and guiding perception for better judgment and interpretation.

### 4.3 Dual-pathway influence of priming on creativity

The study hypothesized that the positively valenced raga would catalyze creativity and elicit greater creative performance than the negatively valenced music and the control cohort. The results of the Alternative Uses Task (AUT) suggested a significant influence of ragas on the creative performance. Priming effects of Raga Kedar consistently enhanced creativity scores across conditions, with participants displaying significantly greater fluency, flexibility and originality than Raga Charukeshi and the control group. This suggests that Raga Kedar, being positive music, is a greater facilitator of divergent thinking and has an effect on the cognitive processes. On the other hand, Raga Charukeshi, being associated with a more somber emotional tone, also displayed a significant priming effect in increasing the creativity performance than the control group, but it was less significant than the positive music. This indicates that although both ragas increased creativity, the degree of impact depends on the emotional valence of music.

These findings are substantiated empirically by earlier research, though these studies utilized background music, rather than priming through music. Positive or “happy” background music fosters divergent thinking (Ritter and Ferguson, 2017) and music-feedback exercise specifically improved performance on the Alternative Uses Task (Fritz et al., 2020). Research by Hosseini et al. (2019) also found an enhanced creativity performance on AUT after exposure to positive background music conditions relative to the no music condition. These papers and existing literature on creativity utilized background music to augment creativity, but the present research employed affective priming through music, thereby addressing a key research gap. Existing research on priming has used other priming media to study its effects on creativity, as Lewis et al. (2011) indicated that affective computational priming through digital images enhanced the creative performance of participants on the Alternative Uses Task, suggesting an unconscious influence via emotional cues on the creative idea generation, broadly supporting the unconscious impact of affective priming in the present study.

As both positively and negatively valenced music enhanced creativity, this pattern aligns with the dual-pathway model of creativity (Xiao et al., 2023), where the priming effects of Raga Kedar broadened the attentional scope, leading to enhanced cognitive flexibility, and Raga Charukeshi fostered creativity by narrowing the attentional focus, which in turn preserved the cognitive resources and maintained moderate arousal. “Broaden-and-Build theory” by Fredrickson (2001) also supports this hypothesis that positive emotions broaden the cognitive repertoires of the individual, which helps them in generating original and innovative ideas, thereby fostering higher creative output.

While ragas in the present study were found to be robust affective primes for fostering creativity, it is imperative to assess these results against the background of broader literature, which reported null or tenuous effects of music on creativity. In their respective research, Yamada and Nagai (2015) and Ritter and Ferguson (2017) pinpointed that music, through mood induction, enhances divergent thinking by testing performance on AUT, but none of its effects were incurred on convergent thinking. Nevertheless, the present study did not test convergent thinking, but only divergent thinking; yet this draws our attention to an important aspect of music’s capability to enhance divergent thinking, which is conducive to the exploration and generation of novel ideas. Bringing this to the fore, Hooi and Tan (2024) and Xia et al. (2023) expounded that music failed to enhance creativity, both divergent and convergent thinking. It is vital to acknowledge, however, that both these studies used background music to bolster creativity. This research addressed the limitation of background music, which has been shown to disrupt the performance on creativity tests due to auditory distraction caused by the semantic content, as it competes with the attentional and working memory resources (Threadgold et al., 2019). Therefore, by incorporating priming through music, that too instrumental music, enabled this research to extract only the potent effects of music rather than being an auditory distraction.

The results of this study revealed a consistent significant effect based on gender differences, meaning that even without any intervention, females outperformed the male participants in the creativity tests, scoring higher in fluency, flexibility and originality. Recent research providing evidence across 62 countries with nearly half a million participants claimed that female participants consistently outperformed male participants in creativity thinking test scores (Goecke et al., 2025). The findings of the present research provide a practical use in educational work, clinical and therapeutic settings, where incorporating music of positive and negative valence can help foster divergent thinking, problem-solving skills, cognitive flexibility and innovation for new ideas.

### 4.4 Dual-pathway influence of priming on figural creativity

The study hypothesized that the positively valenced raga would catalyze creativity and elicit greater creativity performance than the negatively valenced music and the control cohort. The results of the Torrance Tests of Creative Thinking - Figural suggested a significant influence of ragas on the creativity performance. Similar to the findings of the Alternative Uses Task, both ragas augmented creativity, with Raga Kedar being a greater facilitator of divergent and figural creativity, where the participants displayed significantly greater fluency, flexibility, originality, elaboration and abstraction than Raga Charukeshi and the control group. Raga Charukeshi (sad raga) also displayed a significant priming effect in increasing the creativity performance than the control group, but it was less significant than the positive raga.

The differences in the performance of the experimental and control group on the TTCT-figural can be seen in Figure 7. Figures 7A,C represent the responses of experimental groups Kedar and Charukeshi, respectively. Their unique and diverse responses to the same figural stimuli given to both the experimental and control groups reflect higher originality, fluency, flexibility, richer and distinct elaboration, and creative use of abstract titles. In contrast, Figures 7B,D represent the control group’s responses, which are repetitive and showcase a literal use of the given stimuli, along with overly descriptive abstract titles, which reflect lower originality and restricted figural transformation.

These findings are substantiated empirically in earlier research; for instance, Xia et al. (2023) found that music significantly improves overall design creativity and novelty of design solutions. It was found by Palmiero et al. (2023) that the sadness induced by music enhanced visual artistic creativity in non-artists. After sequential exposure to music, participants’ performance on the creative thinking-drawing production test significantly improved (He et al., 2017). A specialized research study by Hahm et al. (2017) examined the brain functioning while performing Torrance Tests of Creative Thinking – Figural without exposure to any music found primary activation in the right fusiform gyrus, frontal, temporal, parietal, and occipital regions, along with widespread bilateral involvement. This research reveals that performing TTCT-figural activates neural activation of these brain areas, thereby providing a neurocognitive basis for how the affective priming through music may have modulated the performance on this creativity test. All these studies propagate that music enhances creative performance in visual and drawing-based creativity tasks.

Prior literature did not use priming through music to enhance creativity; most of the studies utilized background music to augment creativity. The present research employed affective priming through music, thereby addressing a key research gap. Existing research on priming has used other priming media to study its effects on creativity (Lewis et al., 2011), suggesting an unconscious influence via emotional cues on the creative idea generation, broadly supporting the unconscious impact of affective priming in the present study.

While ragas in the present study were found to be robust affective primes for fostering figural creativity, it is imperative to assess these results against the background of broader literature, which reported null or tenuous effects of music on creativity. This study analyzed emotional valence, but the specific effects of arousal were not distinctly measured. In contrast, He et al. (2017) found in their research that only musical arousal of any valence was a significant mediator to enhance scores on the creative thinking drawing production test. No augmentation of creativity was noted by Hooi and Tan (2024), which included figural tests. These studies serve only as a cautionary backdrop, as they used background music to bolster creativity, which disrupts the performance on creativity tests due to auditory distraction caused by the semantic content (Threadgold et al., 2019), whereas the present study incorporated priming through music.

The practical implications of the results of the study are that positive music like Raga Kedar can be intentionally used in educational and creative settings to enhance divergent thinking and visual problem-solving. For instance, art and design instructors can use ragas to their benefit as they not only have the potential to stimulate idea generation, originality, and elaboration but also can modify the perceptual processing (as was shown in the results of the Emotional Word Selection Task) of any given stimulus, which can give new perspectives during drawing and design exercises. In the same way, it can also be applied in workplaces that require creative output, like advertising, architecture or product design, where ragas can help in fostering innovation and enhancing team brainstorming sessions. Additionally, music can be useful in therapeutic and recreational setups for increasing creative expression and engagement, for propagating cognitive flexibility and emotional well-being. As there were no differences found in the study based on gender or age group, this means that these results are generalizable across males and females of the 25–55 age group.

## 5 Limitations and future directions

The present analysis does not come without its drawbacks. The present study was limited to Indian participants of the same ethnicity in the age group of 25–55 years, which compromises the generalizability of the findings. Future research should conduct a retrospective study with a distinct sample and a more flexible age range. The present study assessed the mood and affect using PANAS before the exposure to musical primes, which primarily focused on emotional valence, but the specific effects of arousal were not distinctly measured or experimentally controlled. Considering the closely intertwined nature of valence and arousal as constructs of affect, isolating their effects from each other remains challenging, and hence, the findings should be interpreted with caution. Future research can study the impact and differences based on arousal, timbre and tempo. Even though the participants were carefully screened to minimize the familiarity bias with Indian classical music and specific ragas used in the study, the effects of subtle broader cultural exposure cannot be entirely ruled out.

The research analyzed the mood only before the experiment, but further research can evaluate it after the lexical choice and creativity tests to accurately measure the mood changes. The research employed only two ragas, but future research can be conducted on more distinct ragas. The study incorporated three measures for analyzing perception and creativity, but future research can include other tests or neuroimaging and physiological measures like fMRI and EEG assessment, to provide further evidence for the research. Instead of playing recorded music, future research can also play live music to see if there is a stronger or different effect, or the music exposure can be increased to longer durations of time or repeated exposure to note any differences. Future research can also include a wider emotional spectrum of ragas (e.g., romantic, devotional, calm) to see the effects of different valence beyond the basic emotions. Further studies can also measure individual differences by examining specific personality traits (e.g., openness to experience, musical training or emotional sensitivity) and their different implications on the results. Most importantly, future research can compare different genres of music together to find their differences in priming influence, like ragas, western classical, jazz, or contemporary music.

## 6 Conclusion

In a groundbreaking investigation, this research delves into a largely unexplored territory of affective priming through Indian ragas and their potential influence on the perception of ambiguous visual stimuli and the enhancement of creativity. As previous research was largely concentrated on utilizing background music, this research incorporated affective priming through music and investigated its influence on perception and creativity, bridging this vital gap. This study is the first to explore the multifaceted relationship between supraliminal affective priming, Indian ragas and their emotional valence - all of which significantly impact perception and creativity. Remarkably, the effectiveness of Indian ragas as affective primes was noted on the perception of ambiguous visual pictures, where the emotional words chosen were congruent with the emotional valence of the music, hence the study provides clear evidence for the top-down processing of perceptual stimuli. Another striking finding was that creativity was not just catalyzed by positively valenced music but also by negatively valenced music, though positive music elicited greater creative performance than the negative music, with experimental cohorts surpassing control cohorts on measures of divergent thinking. This paradox—that negative music also fosters creativity- invites a plausible definition of the dual-pathway model of creativity, which outlines that both positive and negative emotions take distinct pathways for fostering creativity. This inquiry foregrounds that music transcends its role as just a conventional auditory stimulus, but rather harnesses its emotional prowess to influence the perceptual processing of external stimuli and fosters cognitive domains like creative thinking and facilitates the genesis of novel ideas.

## Data Availability

The datasets presented in this article are not readily available because the datasets generated and analyzed during the current study are not publicly available due to participant confidentiality and the conditions of informed consent. The materials used in the study (ambiguous visual stimuli) are provided in the Supplementary material, and all statistical results are reported within the article. Requests to access the datasets should be directed to alishadeen@justustherapy.in.

## Footnotes

1
https://youtu.be/hNn2tBohjxE?si=9YtjyGCpRm4UZeni

2
https://youtu.be/X3GbWOegovU?si=1mcD4e6hQ72aaHPG
