# Supplementary material for: Affective priming through Indian ragas: influence on perception of ambiguous visual stimuli and creativity
Source: Front Psychol. 2026 Jan 29;17:1723673. doi: 10.3389/fpsyg.2026.1723673 (PMC12894212; doi:10.3389/fpsyg.2026.1723673)
Supplement: Supplementary file 1 [file Data_Sheet_1.pdf]

Links to ambiguous pictures used in the Emotional word selection task

Due to licensing restrictions, the links to the images purchased under a Standard License from Adobe Stock Library are provided below.

Name of Image	Adobe File ID	Author	Web Link
continuous line drawing of boy making photos with camera	128826289	One Line Man	https://stock.adobe.com/images/continuous-line-drawing-of-boy-making-photos-with-camera/128826289
continuous line drawing of businessman thinking on white background	120599521	One Line Man	https://stock.adobe.com/images/continuous-line-drawing-of-businessman-thinking-on-white-background/120599521
businessman writing a note - single line drawing	157116687	One Line Man	https://stock.adobe.com/images/businessman-writing-a-note-single-line-drawing/157116687
continuous line drawing of business concept - man before open door of opportunity	173139144	One Line Man	https://stock.adobe.com/images/continuous-line-drawing-of-business-concept-man-before-open-door-of-opportunity/173139144
one line drawing of man grilling barbecue	178589010	One Line Man	https://stock.adobe.com/images/one-line-drawing-of-man-grilling-barbecue/178589010
continuous line drawing of young man talking on mobile phone	132179712	One Line Man	https://stock.adobe.com/images/continuous-line-drawing-of-young-man-talking-on-mobile-phone/132179712
businessman making phone call - continuous line drawing	152529686	One Line Man	https://stock.adobe.com/images/businessman-making-phone-call-continuous-line-drawing/152529686
one line drawing of man working with laptop computer behind desk	178588921	One Line Man	https://stock.adobe.com/images/one-line-drawing-of-man-working-with-laptop-computer-behind-desk/178588921
Continuous line drawing. Woman relaxing with cup of tea. Vector illustration.	171728496	Valenty	https://stock.adobe.com/images/continuous-line-drawing-woman-relaxing-with-cup-of-tea-vector-illustration/171728496
Continuous line drawing. Young Boy Playing His Toy Car. Vector illustration	163681640	Valenty	https://stock.adobe.com/images/continuous-line-drawing-young-boy-playing-his-toy-car-vector-illustration/163681640

Man in fez reading Koran. Continuous different width line drawing vector illustration	179745135	Valenty	https://stock.adobe.com/images/man-in-fez-reading-koran-continuous-different-width-line-drawing-vector-illustration/179745135
one line drawing of two sitting men talking	178592581	One Line Man	https://stock.adobe.com/images/one-line-drawing-of-two-sitting-men-talking/178592581
Continuous line drawing. Man in sleeping pose on pillow. Vector illustration	178712883	Valenty	https://stock.adobe.com/images/continuous-line-drawing-man-in-sleeping-pose-on-pillow-vector-illustration/178712883
continuous line drawing of a guy sitting with laptop computer	130164167	One Line Man	https://stock.adobe.com/images/continuous-line-drawing-of-a-guy-sitting-with-laptop-computer/130164167
Kids with books. Back to school concept.	[no longer available on Adobe]	Valenty	https://creativemarket.com/Valenty/1696709-Kids-with-books.-Back-to-school-concept.
one line drawing of commuters passing by	170087272	One Line Man	https://stock.adobe.com/images/one-line-drawing-of-commuters-passing-by/170087272
one line drawing of man sitting and reading	170087294	One Line Man	https://stock.adobe.com/images/one-line-drawing-of-man-sitting-and-reading/170087294
one line drawing of travelers walking	178592568	One Line Man	https://stock.adobe.com/images/one-line-drawing-of-travelers-walking/178592568
Continuous line drawing. Father and son sitting together. Vector illustration Total editable, choose thickness and place of line	176884710	Valenty	https://stock.adobe.com/images/continuous-line-drawing-father-and-son-sitting-together-vector-illustration-total-editable-choose-thickness-and-place-of-line/176884710
continuous line drawing of youth soccer players	120537731	One Line Man	https://stock.adobe.com/images/continuous-line-drawing-of-youth-soccer-players/120537731
Street musician man playing the violin. Continuous line black and white drawing. Line art vector illustration	168651794	Valenty	https://stock.adobe.com/images/street-musician-man-playing-the-violin-continuous-line-black-and-white-drawing-lineart-vector-illustration/168651794
Boy and girl drawing on paper. Back to school concept. Continuous line	164739887	Valenty	https://stock.adobe.com/images/boy-and-girl-drawing-on-paper-back-to-school-concept-continuous-line-drawing-vector-illustration-on-white-background/164739887

drawing. Vector illustration on white background			
_continuous-line-drawing-template	137418165	One Line Man	https://stock.adobe.com/images/continuous-line-drawing-template/137418165
Continuous line drawing. Abstract portrait of a woman with cup of tea. Vector illustration.	159501133	Valenty	https://stock.adobe.com/images/continuous-line-drawing-abstract-portrait-of-a-woman-with-cup-of-tea-vector-illustration/159501133
Teacher with boy writting on Chalk school board . Continuous line drawing vector illustration back to school	162611086	Valenty	https://stock.adobe.com/images/teacher-with-boy-writting-on-chalk-school-board-continuous-line-drawing-vector-illustration-back-to-school/162611086
Man in fez reading Koran. Continuous line drawing vector illustration	158333266	Valenty	https://stock.adobe.com/images/man-in-fez-reading-koran-continuous-line-drawing-vector-illustration/158333266
one line drawing of group of young people talking	178588907	One Line Man	https://stock.adobe.com/images/one-line-drawing-of-group-of-young-people-talking/178588907
one line drawing of man walking with a phone	178588874	One Line Man	https://stock.adobe.com/images/one-line-drawing-of-man-walking-with-a-phone/178588874
continuous line drawing of team meeting	170078880	One Line Man	https://stock.adobe.com/images/continuous-line-drawing-of-team-meeting/170078880
continuous line drawing of people working on laptop computers	170076371	One Line Man	https://stock.adobe.com/images/continuous-line-drawing-of-people-working-on-laptop-computers/170076371
businessman working on laptop computer - single line drawing	157116752	One Line Man	https://stock.adobe.com/images/businessman-working-on-laptop-computer-single-line-drawing/157116752
Continuous line drawing. Little boy sitting with teddy bear on the white background. Vector illustration	178714978	Valenty	https://stock.adobe.com/images/continuous-line-drawing-little-boy-sitting-with-teddy-bear-on-the-white-background-vector-illustration/178714978
continuous line drawing of travelling businessman walking with r	130315399	One Line Man	https://stock.adobe.com/images/continuous-line-drawing-of-travelling-businessman-walking-with-r/130315399
